# Melatonin Application to *Pisum sativum* L. Seeds Positively Influences the Function of the Photosynthetic Apparatus in Growing Seedlings during Paraquat-Induced Oxidative Stress

**DOI:** 10.3389/fpls.2016.01663

**Published:** 2016-11-04

**Authors:** Katarzyna Szafrańska, Russel J. Reiter, Małgorzata M. Posmyk

**Affiliations:** ^1^Laboratory of Plant Ecophysiology, Faculty of Biology and Environmental Protection, University of LodzLodz, Poland; ^2^Department of Cellular and Structural Biology, University of Texas Health Science Center, San Antonio, San AntonioTX, USA

**Keywords:** carotenoids, chlorophyll fluorescence, hydropriming, melatonin, oxidative stress, paraquat, *Pisum sativum* L.

## Abstract

Melatonin, due to its pleiotropic effects plays an important role improving tolerance to stresses. Plants increase endogenous melatonin synthesis when faced with harsh environments as well as exogenously applied melatonin limits stress injuries. Presented work demonstrated that single melatonin application into the seeds during pre-sowing priming improved oxidative stress tolerance of growing seedlings exposed to paraquat (PQ). PQ is a powerful herbicide which blocks the process of photosynthesis under light conditions due to free radicals excess production, when O_2_ is rapidly converted to $O_{2}^{•–}$ and subsequently to other reactive oxygen species. The parameters of chlorophyll fluorescence [*F*_v_/*F*_m_, *F*_v_/*F*_o_, Rfd, ΦPSII, qP, and non-photochemical quenching (NPQ)] in all variants of pea leaves (derived from control non-treated seeds – C, and those hydroprimed with water – H, and hydroprimed with melatonin water solution 50 or 200 μM – H-MEL50 and H-MEL200, respectively) were analyzed as a tool for photosynthetic efficacy testing. Moreover stability of the photosynthetic pigments (chlorophylls *a, b*, and carotenoids) was also monitored under oxidative stress conditions. The results suggest that melatonin applied into the seed significantly enhances oxidative stress tolerance in growing seedlings. This beneficial effect was reflected in reduced accumulation of $O_{2}^{•–}$ in leaf tissues, preservation of photosynthetic pigments, improved functioning of the photosynthetic apparatus and higher water content in the tissues during PQ-mediated stress. Our findings provide evidence for the physiological role of this molecule and serve as a platform for its possible applications in agricultural or related areas of research.

## Introduction

Melatonin, due to its pleiotropic effects (Reiter et al., 2010; Vriend and Reiter, 2015) can play important roles in improving plant tolerance to adverse conditions. Plants increase endogenous melatonin production when faced with harsh environments (Arnao and Hernández-Ruiz, 2014; Zhang et al., 2015); moreover, exogenously applied melatonin limits stress injuries in plants (Janas and Posmyk, 2013; Bajwa et al., 2014; Meng et al., 2014; Kołodziejczyk and Posmyk, 2016). Melatonin also regulates other physiological processes in plants including seed germination, growth promotion, photoperiodic responses, flower development, root system architecture and senescence delay (Murch and Saxena, 2002; Hernández-Ruiz et al., 2005; Janas and Posmyk, 2013; Wang et al., 2013a,b; Byeon and Back, 2014; Chan and Shi, 2015; Wei et al., 2015). Many of the positive melatonin-induced effects in plants are correlated with its strong anti-oxidative properties, since many processes depend on homeostasis in cell redox-status. Melatonin detoxifies a variety of free radicals (FR) and reactive oxygen species (ROS) (Tan et al., 2014, 2015; Zhang and Zhang, 2014; Manchester et al., 2015). A highly appealing property of this molecule, which distinguishes it from most antioxidants, is that its metabolites also have the ability to scavenge ROS and reactive nitrogen species (RNS). Melatonin generates a free radical scavenging cascade which provides a highly protective defense system; thus, even at low concentrations, melatonin is highly effective in protecting organisms from oxidative stress (Galano et al., 2013; Tan et al., 2014, 2015). Although melatonin acts as a direct free radical scavenger, it also elevated the activities of several antioxidant enzymes which assists in its ability to reduce oxidative damage (Rodriguez et al., 2004; Fischer et al., 2013; Reiter et al., 2015; Bałabusta et al., 2016).

Since high quality of seeds is the basis of crop production, our research has developed effective methods to improve their storage and to protect against harmful factors. In relation to this, exogenous melatonin application into the seeds using different priming methods has proven highly effective (Posmyk et al., 2008, 2009a,b; Janas et al., 2009; Szafrańska et al., 2012, 2013, 2014; Kołodziejczyk et al., 2015).

Seed priming is one of the most effective and cost-efficient methods for seed quality improvement and stress tolerance in plants. This technique is based on controlled seed hydration that induces a particular physiological state in plants (initial steps of germination *sensu stricto*); this process allows the application natural and synthetic compounds into the seeds before their germination. The beneficial effects of seed priming on their resistance to environmental stresses is documented (Jisha et al., 2013). Our data indicate that the positive effects of pre-sowing melatonin application by priming relates not only to seed quality (higher germination and vigor under suboptimal conditions) but also to seedling development, plant growth and product yield (Janas et al., 2009; Posmyk et al., 2009a; Szafrańska et al., 2012, 2013, 2014).

Reactive oxygen species overproduced under different stresses are harmful factors that cause lipid peroxidation, enzyme disturbances and DNA damage. On the other hand, the crucial role of ROS in plant signal transduction is also known. Thus plant’s dilemma is not how to totally eliminate ROS, but how to control them (Considine et al., 2015).

The photosynthetic apparatus is a typical physiological, endogenous source of ROS (Foyer and Shigeoka, 2011). Insufficient energy dissipation during photosynthesis triggers excessive chlorophyll excitation, which initiates a reaction with O_2_ to yield singlet oxygen (^1^O_2_). This ROS is responsible for damage to the photosystem and other systems involved in photosynthesis. The formation of ROS in thylakoid membranes can be also initiated through the univalent reduction of O_2_ to form the superoxide anion radical ($O_{2}^{•–}$) at the donor side of photosystem I (PSI) (Tambussi et al., 2004). Taking into account highly effective antioxidant properties of melatonin and its positive role in plant physiology, its potential role in protecting the photosynthetic apparatus is an obvious study to perform.

$O_{2}^{•–}$ is generated in chloroplasts when leaves are treated with paraquat (PQ), a widely used non-selective herbicide for agricultural crops. PQ is a redox-active molecule, which quickly penetrates through the leaves and blocks photosynthesis by accepting electrons from PSI. This leads the inhibition of ferredoxin reduction resulting in depletion of NADPH and inhibition of CO_2_ fixation/assimilation (Moustaka and Moustakas, 2014; Moustaka et al., 2015). The increased efficiency of electron capture by PQ enhances the linear electron transport rate and production of PQ radicals (PQ^+^) which transfer electrons to O_2_ to produce $O_{2}^{•–}$ (Moustaka and Moustakas, 2014). Plants tolerate overproduction of ROS only if sufficient antioxidant mechanisms are involved. We have postulated that melatonin application could help plants to tolerate oxidative stress during PQ exposure.

In plants the energy absorbed by chlorophyll *a* is utilized/deactivated in three different means: (a) a major portion is used for non-cyclic (ATP and NADPH synthesis) or cyclic (ATP synthesis) electron transfer in photosynthesis, (b) excess energy is dissipated as heat, (c) energy is emitted as light (fluorescence; Iriel et al., 2014). Measuring chlorophyll fluorescence is a great tool in determining the photosynthetic efficiency. The measurement of fluorescence not only perfectly illustrates the reactions of photosystem II (PSII) under different abiotic stresses, but also distinguishes the type of stress to which a plant is subjected. Among many techniques available to study photosynthesis, pulse amplitude-modulated (PAM) fluorometry is widely used as a rapid, sensitive and non-invasive tool for the estimation of inhibition and damage in PSII electron transfer process (Moustaka et al., 2015).

Powerful herbicides disrupt ROS homeostasis in plant cells adversely affecting the process of photosynthesis. The objective of the current study was to test whether the pre-sowing seed treatment with melatonin positively influenced the parameters of chlorophyll fluorescence in pea leaves under oxidative stress triggered by PQ.

## Materials and Methods

### Plant Material

*Pisum sativum* L. seeds provided by TORSSED (Torun, Poland) were hydro-primed with water (H), 50 and 200 μM melatonin/water solutions (H-MEL50, H-MEL200), while non-primed seeds were used as a control (C). To perform seed hydro-priming firstly their initial and final water contents were determined and based on these data the amount of water necessary to achieve appropriate seed moisture content was calculated (Posmyk et al., 2008). The seeds were hydro-primed in closed glass bottles on the STR4 DRIVE rotator (BioCote) at room temp. Portions of water and the aqueous MEL solutions were added at 1-h intervals. This procedure lasted for about 6 h, according to the kinetics of pea seed imbibition at room temperature (these parameters were established experimentally). Next the seeds were air-dried for the subsequent 3 days (time sufficient for the seeds to return to the initial water content) and then used for the experiments.

The seeds were surface sterilized with a fungicide (Thiuram, Organika-Sarzyna, Poland), placed in plastic boxes with cotton wool moistened with distilled water and germinated at 25°C for 3 days. The young seedlings were transplanted into plastic pots filled with sterilized universal soil and pearlite (3:1); they were grown for 21 days in a breeding room at constant temperature of 25°C and a fixed photoperiod (16 h light/8 h dark) with light intensity of 7.7–8.4 μmol m^-2^ s^-1^.

### Paraquat Treatment

Paraquat (PQ, methyl viologen, 1,1′-dimethyl 4,4′-bipyridinium dichloride), obtained from Sigma–Aldrich (Germany), was used to trigger oxidative stress in tissues. Leaf disks 18 mm in diameter were cut from 24-day-old pea plants. Some leaf disks were immediately used for analysis (*T*_0_), and the others were put into Petri dishes filled with 15 ml of 75 μM PQ. They were then placed in a growth chamber (Orbis DATA LOG) with constant light (3.5–3.7 μmol m^-2^ s^-1^), at 25°C and incubated for specified time for different analysis: 2, 4, and 6 h for chlorophyll fluorescence parameters analysis and 24 and 48 h for pigment and relative water contents (RWCs). After these times, leaf disks were nitroblue tetrazolium (NBT) stained to identify $O_{2}^{•–}$ generation. Therefore, leaf disks were removed from the Petri dishes, dried on a paper towels and used for further analysis.

### PAM Fluorometry

Chlorophyll fluorescence parameters were obtained with a pulse amplitude modulated (PAM) fluorometer (JUNIOR-PAM, WALZ Germany), using WinControl Windows Software, according to the manufacturer’s instruction. The leaf disks previously incubated with PQ 75 μM were transferred into Petri dishes containing distilled water and dark adapted for 30 min before the fluorescence measurements started. To record the chlorophyll fluorescence, the leaf disks, supported with a special clip, were illuminated with a modulated beam of low intensity light (ML, 200–300 mV) to measure the initial fluorescence (*F*_0_). Maximal fluorescence (*F*_m_) was determined after exposure to a saturating pulse of white light (SP, 10 000 μmol photons m^-2^ s^-1^, 800 ms) to close all reaction centers. From these data, the maximum photochemical quantum yield of PS II (*F*_v_/*F*_m_, where *F*_v_ = *F*_m_-*F*_0_) and *F*_v_/*F*_0_ (a value that is proportional to the activity of the water-splitting complex on the donor side of the PSII) were calculated. Subsequently, the samples were exposed to the actinic light (AL, 190 μmol photons m^-2^ s^-2^) until a steady-state fluorescence value (*F*_s_) was reached, and a new SP was applied to record the maximum fluorescence for light-adapted leaves (F_m_′). The quantum efficiency of PSII [ΦPSII = (F_m_′-*F*_s_)/F_m_′) was then obtained. The vitality index (Rfd, chlorophyll fluorescence decrease ratio) which is the indicator of CO_2_ fixation was calculated as (*F*_m_-*F*_s_)/*F*_s_ (Lichtenthaler et al., 2005). The photochemical quenching (qP), which quantifies the actual fraction of PSII reaction centers (RCs II) being in the open state was calculated as (F_m_′-*F*_s_)/(F_m_′-F_0_′) (Genty et al., 1989). The NPQ parameter, which was calculated as (*F*_m_-F_m_′)/F_m_′, estimates the NPQ that reflects heat dissipation of excitation energy in the antenna system (Bilger and Björkman, 1990).

All the measurements were performed at room temperature (25°C), in the dark room lit only with dim green light to facilitate work, on the ad-axial face of leaves. Each chlorophyll fluorescence parameter represents mean values from 6 to 7 leaf disks each with 4–5 areas of interest (*n*∼25–30).

### Pigments Content

Chlorophyll *a, b, a*+*b* and carotenoids were quantified spectrophotometrically. The leaf disks (25 mg) were homogenized in a chilled mortar and pestle with MgCO_3_ and 5 ml of 80% acetone and filtered. In the obtained supernatant absorbance at three wavelengths: 470, 646, and 663 nm (spectrophotometer Hitachi U-2001) was measured, which then was used to calculate chlorophyll *a, b, a*+*b* and carotenoid concentrations with the following formulas Lichtenthaler and Buschmann (2001):

$$\begin{array}{c} Chla\;=\;12.25\;\times \;A_{663}\;-\;2.79\;\times \;A_{646}\\ Chlb\;=\;21.50\;\times \;A_{646}\;-\;5.10\;\times \;A_{663}\\ Chla+b\;=\;7.15\;\times \;A_{663}\;+\;18.71\;\times \;A_{646}\\ Carotenoids\;=\;(1000\;\times \;A_{470}\;-\;1.82Chla\;-\;85.02Chlb)/198 \end{array}$$

Pigment assays were performed in at least five replicates (*n* = 5).

### Relative Water Content (RWC)

Determination of the RWC was performed according to Barrs (1968). RWC was calculated as follows: RWC [%] = [(FW–DW)/(SW–DW)] × 100%, where FW is the fresh weight, DW is the dry weight determined after 48 h in an oven at 90°C and SW is the saturated weight measured after 4 h of saturation in deionized water at room temperature in the dark. Experiment was performed in five replicates (five leaf disks per each) (*n* = 5).

### Nitroblue Tetrazolium (NBT) Staining for *Pisum sativum* L. Leaves

Location of $O_{2}^{•–}$ in pea leaf disks was performed by NBT staining according to the method of Kawai-Yamada et al. (2004). All leaf disks were investigated at time points: *T*_0_, 2, 4, 6, 24, and 48 h of incubation in PQ 75 μM. The plant material was first infiltrated with 10 mM NaN_3_ solution in potassium phosphate buffer (pH 7.8), then with 0.1% (v/v) NBT solution in potassium phosphate buffer (pH 7.8). Subsequently it was incubated for 2 h with NBT solution under light at room temp. After this time, the leaf disks were transferred into wide tubes and boiled in AGE solution (acetic acid: glycerol: ethanol (1: 1: 3 [v/v/v])), up to discolouration of chlorophyll. The stained disks were transferred onto Petri dishes, analyzed using Binocular – Hund-WETZLAR, and then photographed.

### Superoxide Dismutase Extraction and Assay

Protein extraction was performed according to Bałabusta et al. (2016). One gram of leaf disks was ground in a mortar and homogenized with 0.5 g PVP in 5 mL of 0.1 M phosphate buffer (pH 7.5) containing 2.5 mM DTT, 1 mM EDTA, 1.25 mM PEG-4000, and 1 mM PMSF. The homogenate was centrifuged at 20000 *g* for 30 min at 4°C. The obtained supernatant was filtered through Miracloth, desalted on a PD10 column (Pharmacia, Uppsala, Sweden) and used for the enzyme assays. All steps of the extraction procedure were carried out at 4°C.

Superoxide dismutase (EC1.15.1.1) activity was measured according to Giannopolitis and Ries (1977). The reaction mixture contained 2 mM riboflavine, 13 mM methionine, 0.1 mM EDTA, 70 mM NBT in 0.1 M phosphate buffer (pH7.5), and 100 ml of the enzyme extract in the final volume of 3 ml. SOD activity was assayed by measuring the ability of the enzyme to inhibit the photochemical reduction of NBT. Glass test tubes containing the mixture were illuminated with a fluorescent lamp at 25°C (Philips MLL 5000W, Eindhoven, The Netherlands). Identical tubes, which were not illuminated served as blanks. After illumination for 15 min, absorbance was measured at 560 nm. One unit of SOD was defined as the enzyme activity, which inhibited the photoreduction of NBT to blue formazan by 50%. SOD activity was expressed as the enzyme unit per milligram of protein (U mg^-1^ prot.).

### Statistical Analyses

The results represent the average values ± standard error (±SEM) of the mean. The data were analyzed using STATISTICA v.10.0_MR1_PL [StatSoft] software. The two-way analysis of variance (ANOVA) and then the *post hoc* Duncan multiple range tests were carried out to find the significant differences at *p* < 0.001 in each experiment.

## Results

**FIGURE 1:**
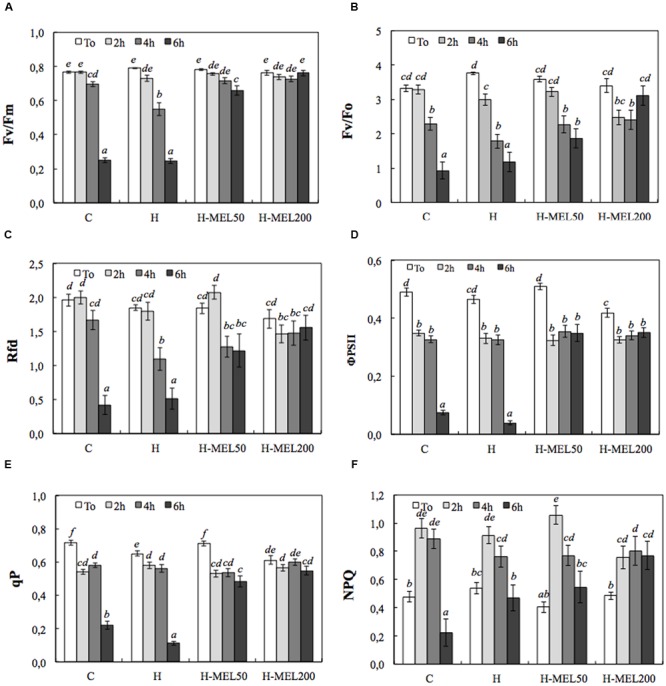
**Chlorophyll fluorescence parameters of leaf disks cut out of 24-day-old pea plants grown from the control (C), hydroprimed (H), and hydroprimed with an aqueous solution of 50 μM melatonin (H-MEL50) or 200 μM melatonin (H-MEL200) seeds.** Measurements were performed at *T*_0_ (before PQ treatment) and after 2, 4, and 6 h of disk incubation in 75 μM PQ solution. Individual graphs show: **(A)** maximum photochemical quantum yield of PSII in the dark-adapted state (*F*_v_/*F*_m_); **(B)** the efficiency of the water-splitting complex on the donor side of PSII (*F*_v_/*F*_0_); **(C)** vitality index (Rfd); **(D)** quantum efficiency of PSII (ΦPSII); **(E)** photochemical quenching (qP), **(F)** non-photochemical quenching (NPQ). The results are expressed as mean values of about 25 measurements ± SEM. Two-way ANOVA and Duncan’s *post hoc* test were performed. The small letters on the graphs show statistical significance. ANOVA results: ***F*_v_/*F*_m_ –** Variant (C, H, H-MEL50, and H-MEL200) *F*_(3,331)_ = 87.4 *p* < 0.0001; Time (*T*_0_, 2, 4, and 6h) *F*_(3,331)_ = 217 *p* < 0.0001; and interaction Variant × Time *F*_(9,331)_ = 53.4 *p* < 0.0001; ***F*_v_/*F*_0_ –** Variant (C, H, H-MEL50, and H-MEL200) *F*_(3,376)_ = 3.77 *p* < 0.01; Time (*T*_0_, 2, 4, and 6 h) *F*_(3,376)_ = 52.2 *p* < 0.0001; and interaction Variant × Time *F*_(9,376)_ = 7.2 *p* < 0.0001; **Rfd –** Variant (C, H, H-MEL50, and H-MEL200) *F*_(3,359)_ = 3.31 *p* < 0.01; Time (*T*_0_, 2, 4, and 6 h) *F*_(3,359)_ = 36.5 *p* < 0.0001; and interaction Variant × Time *F*_(9,359)_ = 6.2 *p* < 0.0001; **ΦPSII –** Variant (C, H, H-MEL50, and H-MEL200) *F*_(3,296)_ = 27.4 *p* < 0.0001; Time (*T*_0_, 2, 4, and 6 h) *F*_(3,296)_ = 152.2 *p* < 0.0001; and interaction Variant × Time *F*_(9,296)_ = 24.1 *p* < 0.0001; **qP –** Variant (C, H, H-MEL50, and H-MEL200) *F*_(3,296)_ = 21.3 *p* < 0.0001; Time (*T*_0_, 2, 4, and 6 h) *F*_(3,296)_ = 156 *p* < 0.0001; and interaction Variant × Time *F*_(9,296)_ = 25.9 *p* < 0.0001; **NPQ –** Variant (C, H, H-MEL50, and H-MEL200) *F*_(3,294)_ = 0.66 *p* = 0.5; Time (*T*_0_, 2, 4, and 6 h) *F*_(3,294)_ = 38.1 *p* < 0.0001; and interaction Variant × Time *F*_(9,294)_ = 4.27 *p* < 0.0001.

Tolerance of studied pea leaves to PQ was evaluated on the basis of their reaction to the first 6 h of PQ treatments. In all investigated variants the *F*_v_/*F*_m_ value did not exceed 0.8 at *T*_0_ (**Figure 1A**). After 6 h of PQ incubation, a sharp decline in this parameter was observed in C and H leaf disks (by about 70%), whereas in H-MEL50 and H-MEL200 leaves, *F*_v_/*F*_m_ ratio remained high, representing 85% and almost 100% of *T*_0_ value, respectively.

**Figure 1B** shows the *F*_v_/*F*_0_ ratio which is more sensitive than the *F*_v_/*F*_m_. In C, H, and H-MEL50 leaves, the decline of this parameter started after 2 h after PQ treatment and gradually progressed to 6 h; however, in H-MEL50 leaves, this decline was not as rapid (**Figure 1B**). After 6 h of PQ incubation, *F*_v_/*F*_0_ ratio in H-MEL200 leaves, was even higher than after 2 and 4 h of treatments, and was only 8.5% lower than that at *T*_0_.

The profiles of the chlorophyll fluorescence decline ratio (Rfd) and the *F*_v_/*F*_m_ changes were similar in C, H, and H-MEL200, while in H-MEL50 after 2 h of PQ treatment Rfd slightly increased (by 13%), after 4 h decreased (by 30%) and after 6 h it remained stable (**Figure 1C**). The differences between melatonin untreated variants (C and H) and those treated with melatonin (H-MEL50 and H-MEL200) after 6 h of PQ incubation were statistically significant.

The quantum efficiency of PSII (ΦPSII), decreased by about 30% in all studied variants after 2 h of PQ treatment and although the additional hours of incubation triggered drastic an ETR decline in C and H leaf disks (by about 90%), in the variants treated with melatonin it remained at the same level (**Figure 1D**).

The tendencies of photochemical quenching (qP) changes were similar; in the C and H they sharply decreased after 6 h of PQ incubation (relatively to *T*_0_ by about 70 and 90%, respectively), whereas in H-MEL50 and H-MEL200 it remained at a high level (decreased only by about 30 and 10%, respectively) (**Figure 1E**). These differences were statistically significant.

The NPQ exhibited a significant increase after 2 h of PQ treatment in all studied variants and in H-MEL50 it reached 260% of *T*_0_ value (**Figure 1F**). Along with the prolonged time of PQ incubation NPQ levels decreased and after 6 h in C and H leaves were lower than at *T*_0_, but in H-MEL50 and H-MEL200 leaves this decline was less pronounced.

**FIGURE 2:**
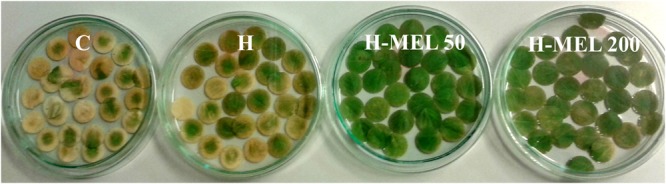
**Changes in green color intensity of leaf disks cut out of 24-day-old pea plants grown from the control (C), hydroprimed (H), and hydroprimed with an aqueous solution of 50 μM melatonin (H-MEL50) or 200 μM melatonin (H-MEL200) seeds.** Photographs were taken after 48 h of disks incubation in 75 μM PQ solution. The content of leaf pigments with statistical analysis at this time point (48 h) is presented in **Figure 3** as dark gray bars.

**FIGURE 3:**
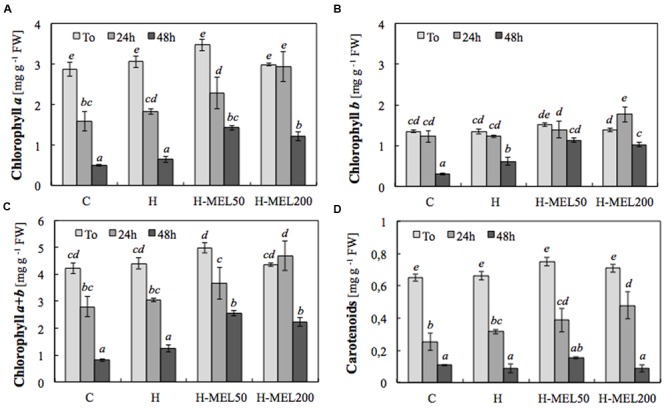
**Contents of chlorophyll *a***(A)**, *b***(B)**, *a*+*b***(C)** and carotenoids **(D)** in leaf disks cut from 24-day-old pea plants grown from the control (C), hydroprimed (H), and hydroprimed with an aqueous solution of 50 μM melatonin (H-MEL50) or 200 μM melatonin (H-MEL200) seeds.** Measurements were performed at *T*_0_ (before PQ treatment) and after 24 and 48 h of disk incubation in 75 μM PQ solution. The results are expressed as mean values of about 5 measurements ± SEM. Two-way ANOVA and Duncan’s *post hoc* test were performed. The small letters on the graphs show statistical significance. ANOVA results: **Chlorophyll *a* –** Variant (C, H, H-MEL50, and H-MEL200) *F*_(3,24)_ = 11.6 *p* < 0.0001; Time (*T*_0_, 2, 4, and 6 h) *F*_(2,24)_ = 127 *p* < 0.0001; and interaction Variant × Time *F*_(6,24)_ = 2.68 *p* < 0.05; **Chlorophyll *b* –** Variant (C, H, H-MEL50, and H-MEL200) *F*_(3,24)_ = 12.8 *p* < 0.0001; Time (*T*_0_, 2, 4, and 6 h) *F*_(2,24)_ = 51 *p* < 0.0001; and interaction Variant × Time *F*_(6,24)_ = 3.91 *p* < 0.01; **Chlorophyll *a*+*b* –** Variant (C, H, H-MEL50, and H-MEL200) *F*_(3,24)_ = 12.6 *p* < 0.0001; Time (*T*_0_, 2, 4, and 6 h) *F*_(2,24)_ = 97.6 *p* < 0.0001; and interaction Variant × Time *F*_(6,24)_ = 2.84 *p* < 0.05; **Carotenoids –** Variant (C, H, H-MEL50, and H-MEL200) *F*_(3,24)_ = 4.31 *p* < 0.01; Time (*T*_0_, 2, 4, and 6 h) *F*_(2,24)_ = 218 *p* < 0.0001; and interaction Variant × Time *F*_(6,24)_ = 1.78 *p* = 0.14.

To obtain significant differences in chlorophyll and carotenoid contents, PQ incubation time was extended to 24 and 48 h. Treatment of the leaves with 75 μM PQ significantly influenced their color intensity but even if after 24 h the differences between variants were hardly visible (data not shown); 48 h of PQ incubation resulted in nearly 100% discoloration of C leaf disks with slightly less depigmentation of H leaves. In H-MEL50 leaves the green color was almost completely preserved (**Figure 2**). This visual observation was confirmed by biochemical assays. After 24 h of PQ treatment the chlorophyll *a* level was reduced in C, H, and H-MEL50 leaves by 45, 40, and 34%, respectively; in the H-MEL200 leaves almost no change was observed. In all investigated variants prolonged PQ stress (48 h) caused a significant reduction in the chlorophyll *a* content, but in H-MEL50 it was the greatest and almost 2.5 times greater than in C leaves (**Figure 3A**). These differences were statistically significant. The content of chlorophyll *b* was much lower than that of chlorophyll *a* and 24 h of PQ treatment triggered only a slight decrease in C, H, and H-MEL50. In H-MEL200 this parameter even increased by 28%. A significant reduction in chlorophyll *b* level was observed after 48 h, especially in C and H leaves, while in the variants treated with melatonin it was still relatively high (**Figure 3B**). The schema of carotenoid content changes was similar. Twenty-four hours of PQ incubation caused significant reductions in all variants, and the highest level being preserved in H-MEL200 leaves. After 48 h of PQ treatment, this decline was dramatic but finally the highest level of carotenoids was noted in H-MEL50 leaves (**Figure 3D**).

The RWC was higher in the leaves derived from melatonin primed seeds even before they were transferred to the PQ solution (*T*_0_) (**Table 1**). After 24 h of PQ treatment in C, H, H-MEL50, and H-MEL200 leaves these values rose by about 16, 15, 17, and 20%, respectively. Fourty-eight hours of PQ stress caused RWC reduction in all investigated variants, but it remained the lowest in the C.

**Table 1:** Relative water content (RWC) in leaf disks of 24-day-old pea plants grown from the control (C), hydroprimed (H), and hydroprimed with an aqueous solution of 50 μM melatonin (H-MEL50) or 200 μM melatonin (H-MEL200) seeds.

	RWC (%)			
Variant	C	H	H-MEL50	H-MEL200
**Time**				
*T*_0_	90.4 ± 0.36^a^	90.8 ± 1.36^a^	92.3 ± 1.75^a^	91.9 ± 0.88^a^
24 h	102.9 ± 4.02^c^	104.0 ± 1.74^c^	108.6 ± 3.73^d^	110.0 ± 3.89^d^
48 h	98.5 ± 2.68^b^	105.8 ± 2.30^cd^	102.5 ± 0.75^c^	104.8 ± 2.88^c^

*Measurements were performed at *T*_0_ (before PQ treatment) and after 24 and 48 h of disk incubation in 75 μM PQ solution. The results are expressed as mean values of about 5 measurements ± SEM. Two-way ANOVA and Duncan’s *post hoc* test were performed. The small letters next to the values show statistical significance. **RWC** ANOVA results: Variant (C, H, H-MEL50, and H-MEL200) *F*_*(3;32)*_ = 9.11 *p* < 0.0002; Time (*T*_0_, 2, 4, and 6 h) *F*_*(2;32)*_ = 166 *p* < 0.0001; and interaction Variant × Time *F*_*(6;32)*_ = 3 *p* < 0.02.*

**FIGURE 4:**
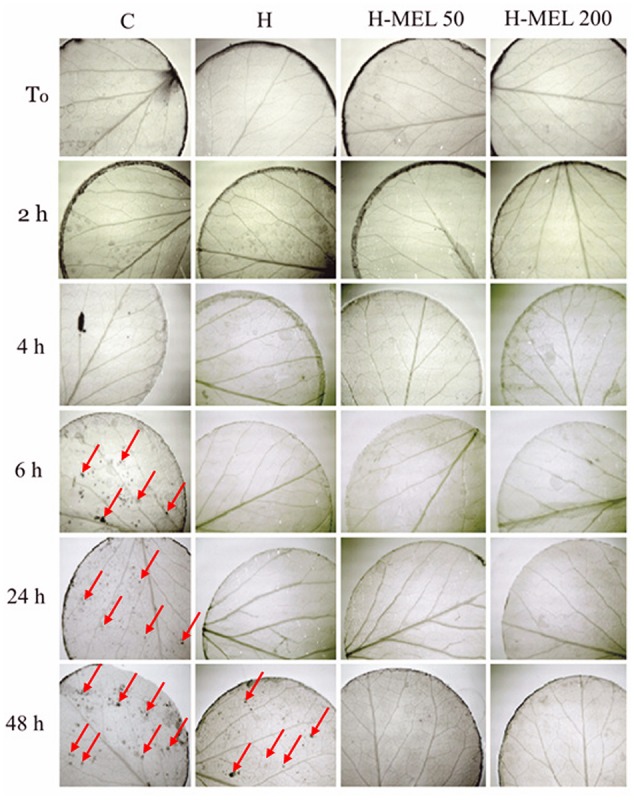
**Detection of $O_{2}^{•–}$ in leaf disks of 24-day-old pea plants grown from the control (C), hydroprimed (H), and hydroprimed with an aqueous solution of 50 μM melatonin (H-MEL50) or 200 μM melatonin (H-MEL200) seeds.** Photographs were taken at *T*_0_ (before PQ treatment) and after 2, 4, 6, 24, and 48 h of disk incubation in 75 μM PQ solution. Red arrows indicate dark spots of formazan derived from NBT oxidized by $O_{2}^{•–}$.

To visualize $O_{2}^{•–}$ in leaf tissues, NBT staining was used. At the starting point (*T*_0_) and for the first 2 h of PQ treatment this anion was located only on the rim of the leaf disks as the result of mechanical injury (**Figure 4**). After 6 h of PQ stress the ROS appeared inside the C disks, while in the other variants, they were still not present. Even 24 h of PQ incubation did not change this status and only after 48 h $O_{2}^{•–}$ appeared also inside H disks. In H-MEL50 and H-MEL200 leaves throughout the experiment the presence of $O_{2}^{•–}$ was not detected (**Figure 4**).

**FIGURE 5:**
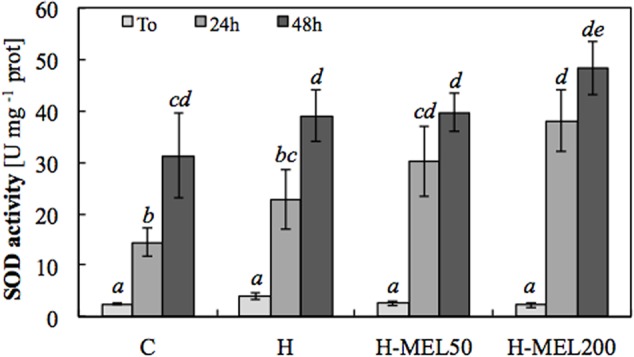
**Superoxide dismutase (SOD) activity in leaf disks cut from 24-day-old pea plants grown from the control (C), hydroprimed (H), and hydroprimed with an aqueous solution of 50 μM melatonin (H-MEL50) or 200 μM melatonin (H-MEL200) seeds.** Measurements were performed at *T*_0_ (before PQ treatment) and after 24 and 48 h of disk incubation in 75 μM PQ solution. The results are expressed as mean values of about 6–9 measurements ± SEM. Two-way ANOVA and Duncan’s *post hoc* test were performed. The small letters on the graphs show statistical significance. ANOVA results: **SOD –** Variant (C, H, H-MEL50, and H-MEL200) *F*_(3,75)_ = 5.51 *p* < 0.001; Time (*T*_0_, 24 and 48 h) *F*_(2,75)_ = 90.7 *p* < 0.0001; and interaction Variant × Time *F*_(6,75)_ = 2.12 *p* < 0.05.

The lack of $O_{2}^{•–}$ accumulation in the leaves of plants grown from the seeds pre-treated with melatonin was probably due to the elevated SOD activity in these plants (**Figure 5**). Under oxidative stress induced by PQ, SOD activity increased in all experimental variants, but the highest was in H-MEL50 and H-MEL200 leaf disks (**Figure 5**), where $O_{2}^{•–}$ was not revealed (**Figure 4**).

## Discussion

Long-term exposure of plants to melatonin before or during stress is a common popular means to verify the impact of exogenous melatonin application on defense mechanisms activated under harmful environmental conditions. There are several reports on such melatonin application and its influence on protein and photosynthetic pigment degradation during aging (Wang et al., 2012, 2013a,b; Liang et al., 2015). However, there is little information concerning single application of melatonin to seeds and its effect on subsequent processes that occur in plants grown from these seeds. Some publications shown that the positive priming effects that occur when beneficial compounds are applied to the seeds are not only visible during germination and sprouting, but also prolonged into later plant developmental stages and observed in more mature plants, especially when subjected to different environmental stresses. There are suggestions that seed conditioning may be a method of improving tolerance through ‘priming memory’ (Savvides et al., 2016). Generally it is provoked by induction of stress reaction syndrome without negative metabolic disorders and injuries, e.g., through epigenetic modifications and/or synthesis of corresponding anti-stress proteins (Kołodziejczyk et al., 2016a,b).

Considering the well-known antioxidant properties of melatonin (Zhang and Zhang, 2014; Manchester et al., 2015) we tested if this molecule, applied into the seeds, could protect the photosynthetic apparatus of growing seedlings against oxidative stress generated in the 24-day-old pea leaves by 75 μM PQ. Due to potent action of PQ for blocking the photosynthesis process, its influence on the chlorophyll fluorescence parameters has been widely investigated; these parameters are highly sensitive indicators of stress intensity (Guo et al., 2007; Rodriguez et al., 2012; Iriel et al., 2014; Moustaka and Moustakas, 2014; Moustaka et al., 2015). Herein, we performed PAM analyses after leaves were incubated for 2, 4, or 6 h with PQ. Generally, the analysis of photosynthesis parameters allows the estimation of plant susceptibility or tolerance to stress.

The *F*_v_/*F*_m_ ratio reflects the maximum photochemical quantum yield of PSII; values between 0.75 and 0.85 in non-stressed plants are considered normal (Silva et al., 2014). This is an important coefficient that indicates how efficiently the light reactions proceed. A rapid decline in *F*_v_/*F*_m_ ratio in the plants that were not pre-treated with melatonin was observed not only in our leaf disks, as shown here, but also in tomato and maize plants subjected to drought stress (Liu et al., 2015; Ye et al., 2016), in detached apple leaves during dark- and drought-induced senescence (Wang et al., 2012, 2013b) and in cucumber plants under salinity stress (Wang et al., 2016); in tissues treated with exogenous melatonin this ratio always remains high. It may be that melatonin improves the rate of electron transport and the efficiency of photochemical conversion (Liu et al., 2015). If the decline in *F*_v_/*F*_m_ is due to photoinhibition of PSII units or to other causes it is a less reliable index. Since *F*_v_/*F*_m_ is a relatively inert and measured in a dark-adapted state, stress-induced changes are detected rather late. The *F*_v_/*F*_0_ ratio is generally more sensitive, since it expresses the efficiency of the water-splitting complex on the donor side of PSII, which is the most sensitive component in the photosynthetic electron transport chain. This ratio includes the same basic information but exhibits higher values and a higher dynamic range than the *F*_v_/*F*_m_ (Lichtenthaler et al., 2005). This is consistent with the current findings which show that changes in *F*_v_/*F*_0_ ratio are more rapid and greater than the *F*_v_/*F*_m_ ratio, especially in leaves treated with MEL50. Usually a reduction in *F*_v_/*F*_0_ parameter results from photosynthetic electron transport impairment (Pereira et al., 2000); this is consistent with our results. This more sensitive ratio under stress conditions (PQ treatment) remained at the higher level in the plant variants pre-treated with melatonin (H-MEL50 and H-MEL200).

The vitality index (Rfd), which represents the chlorophyll fluorescence decline ratio is another parameter that complements the information regarding the PSII photochemistry. The omission of this factor often leads to false conclusions about the function of the photosynthetic apparatus. Under various stress conditions, Rfd value markedly decreases even if no changes in the *F*_v_/*F*_m_ ratio is observed. This may indicate a decline in photosynthetic quantum conversion. In the present work, we observed that *F*_v_/*F*_m_ ratio was very high in the melatonin treated variants, but Rfd of these leaves began to decline during PQ-mediated stress. At the end of the experiment, however, the Rfd index was still higher in H-MEL50 and H-MEL200 leaves than in the C and H leaves; this confirms the positive effect of melatonin on the function of the photosynthetic apparatus under PQ-induced oxidative stress. Additionally, the persistence of high ΦPSII in the melatonin-treated variants after 6 h of PQ-incubation is consistent with this conclusion. Similarly, a higher ΦPSII in melatonin pre-treated apple leaves subjected to drought stress were observed by Wang et al. (2013b) and Ye et al. (2016).

In the current study, the lower quantum efficiency of PSII (ΦPSII), and photochemical quenching (qP) was accompanied by an elevation in NPQ, especially after first 2 and 4 h of PQ-treatment. Changes in qP, that indicates the amount of PSII reactive centers (RCs), that are open, are due to saturation of photosystem with light which results in closure of RCs. A significant decrease of qP in C and H leaves caused by 6 h of PQ treatment indicates that this herbicide reduced the number of open PSII centers; after melatonin pre-treatment, the both leaf variants (H-MEL50 and H-MEL200) had a greater capacity for photochemical quenching under oxidative stress.

Non-photochemical quenching reflects heat dissipation of excitation energy in the antenna system and serves as a photoprotective mechanism. It is related to proton concentrations inside thylakoids and induces the quenching of thermal energy through the xanthophylls cycle (Jahns and Holzwarth, 2012). This cycle transforms the excitation energy into heat and thereby prevents the formation of harmful ROS (Wang et al., 2010). After 2 h of PQ treatment in all studied variants, only a small qP decline occurred, whereas NPQ exhibited a significant increase. This demonstrates that qP, as a measure of the fraction of open PSII RCs, changes little under short term PQ stress; a significantly increased NPQ suggests that the prevailing processes causing the fluorescence quenching are of a photoprotective nature (Serôdio and Lavaud, 2011). During induced apple leaf senescence, a gradual increase in NPQ levels was also observed, but when the plants were treated with melatonin they dropped significantly. This was in accordance with the relatively high rate of photosynthesis and ΦPSII (Wang et al., 2013b). Similar effects were observed in cucumber leaves treated with melatonin and subjected to PEG stress (Zhang et al., 2013).

Fluorescence measurements, in tandem with photosynthetic pigment level analysis, leads to a more complete understanding of the energy dissipation pathways at the RCs of PSII and the pigment-light harvesting complexes. It is known that degradation of photosynthetic pigments is closely linked to the aging process. The precise function of melatonin in delaying leaf senescence in plants remains largely undefined, although remarkable advances have been made in understanding its role *in vivo.* The majority of previous studies ware focused on positive effect of melatonin in senescence process induced by different stress factors. Studies on salt-stressed rice discovered that melatonin treatment significantly reduced chlorophyll degradation, delayed leaf senescence, and enhanced salt stress tolerance (Liang et al., 2015). Melatonin suppressed expression of four senescence-associated genes involved in chlorophyll degradation [stay-green (SGR), non-yellow coloring 1 (NYC1) and 3 (NYC3) genes, and red chlorophyll catabolite reductase 1 (RCCR1)], as well as four senescence-induced genes (OsNAP, Osh36, Osh69, and OsI57), which are widely used as age-dependent or dark-induced leaf senescence markers in rice (Liang et al., 2015). Soybean seed-coating with melatonin improved their tolerance to salt and drought stress, probably due to enhanced expression of genes related to photosynthesis, carbohydrate/fatty acid metabolism, and ascorbate biosynthesis (Wei et al., 2015). In these studies melatonin upregulated two subunits of photosystem I (PS I) (PsaK and PsaG), two elements (PsbO and PsbP) related to the oxygen-evolving complex of PS II (oxygen-evolving enhancer proteins), the ferredoxin gene PetF, and the VTC4 gene, encoding the L-galactose 1-P-phosphatase involved in ascorbate biosynthesis (Wei et al., 2015). Dark-induced senescence of apple leaves was also inhibited by exogenous melatonin application (Wang et al., 2012). Melatonin delayed the normal chlorophyll degradation and reduced the decline of *F*_v_/*F*_m_ ratio. It also suppressed the transcript levels of a key chlorophyll degradation gene, pheide a oxygenase (PAO), and the senescence-associated gene 12 (SAG12). The slower process of protein degradation during apple leaf senescence was also noticed, probable as a result of melatonin-linked inhibition on the expression of autophagy-related genes (ATGs) (Wang et al., 2013a). Additionally, in melatonin-treated plants the expression of genes encoding the small subunit of Rubisco (RBCS), and proteins binding chlorophyll a/b (CAB), was inhibited much more slowly than in the control non-treated group. Moreover, in these plants the process of photosynthesis was more efficient, and concentrations of sucrose, starch and sorbitol were higher (Wang et al., 2013a). In cucumber plants under salinity stress, the addition of melatonin efficiently alleviated the decrease in the net photosynthetic rate, the maximum quantum efficiency of PSII, and the total chlorophyll content (Wang et al., 2016). Additionally, melatonin enhanced the activity of antioxidant enzymes (including SOD, POD, CAT, and APX) and concentrations of antioxidants (ascorbic acid and glutathione), reducing in this way the oxidative damage and increasing salinity tolerance of plants. Natural senescence of *Arabidopsis thaliana* leaves was also delayed by exogenous melatonin treatment and one positive regulator of natural leaf senescence -AtIAA17 [gene of auxin-resistant 3 (AXR3)/indole-3-acetic acid–inducible 17] was significantly repressed (Shi et al., 2015). Transcriptome analysis of *A. thaliana* suggests that melatonin may play critical role(s) in plant defense systems (Weeda et al., 2014). Authors discovered that out of nearly 900 genes that were significantly up- or down- regulated by melatonin with at least twofold changes, almost 40% of them were related to plant stress defense, including many stress receptors, kinases and transcription factors, as well as downstream genes encoding end products that were directly used for stress defense. In the presented work melatonin supported chlorophyll preservation during 48 h of PQ-induced stress, and although the mechanism of its action is not fully explored, it could indicates that melatonin pre-treatment enhances synthesis and/or slows down decomposition of chlorophyll under oxidative stress.

Carotenoids also function as accessory pigments and act as photo-protectants and serves as safety valves releasing excess energy before it can damage plant cells (Shumskaya and Wurtzel, 2013). In this study, carotenoids in tandem with chlorophylls seem to aid melatonin pre-treated plants to counteract PQ-induced stress. However, when a very high dose of herbicide was applied, even increasing the carotenoid content was not sufficient to prevent injuries and the consequential death, as was shown in fodder radish plants (Silva et al., 2014).

Paraquat as a fast-acting herbicide directly penetrates leaves and within a few hours can lead to death of plants due to loss of water (Ekmekci and Terzioglu, 2005; Watts, 2011). To determine plant tissue hydration, RWC is usually measured (Zhang et al., 2013). In the present study, melatonin application to pea seeds resulted in rise of RWC in the leaves under PQ-induced oxidative stress. In contrast, C leaf disks revealed a significant loss of water after 48 h of PQ exposure, even when they were incubated in an aqueous medium. These results indirectly testify to the loss of membrane integrity in C plants and to the maintenance membrane integrity in H-MEL50 and MEL200 plants. This finding verifies that melatonin enrichment of seeds significantly inhibited oxidative damage to membrane under PQ-generated stress. A similar effect was observed under drought stress in *Malus* species pre-treated with melatonin (Li et al., 2015). According to these authors, water status can be improved by dual protective mechanisms (reduced contents of both ABA and H_2_O_2_) working synergistically to improve stomata functioning. A mitigating effect of melatonin on RWC was also found in wheat seedlings under cold stress (Turk et al., 2014), although in soybean plants subjected to drought stress, RWC was slightly lower than in the control plants (Wei et al., 2015).

Paraquat is a herbicide which blocks the process of photosynthesis under light conditions due to free radicals excess production, when O_2_ is rapidly converted to $O_{2}^{•–}$ and subsequently to other ROS. NBT staining did not reveal $O_{2}^{•–}$ in the tissues of H-MEL50 and H-MEL200 leaves which confirms the radical scavenging activity of melatonin under PQ-stress. These observations are consistent with the results of Hasan et al. (2015), whose histochemical study showed reduced levels of ROS in tomato leaves pre-treated with melatonin under Cd stress. They also demonstrated a significant ameliorative effect of melatonin on *F*_v_/*F*_m_ ratio and net photosynthesis rate during Cd stress.

The analysis of SOD activity, the key enzyme regulating the $O_{2}^{•–}$ status in leaves, confirmed the positive indirect effect of melatonin on this radical elimination. Usually SOD is activated in the face of oxidative stress, which can be clearly seen here in all experimental variants at 24 and 48 h of PQ treatment. However, the highest, statistically important increase in SOD activity was noted in H-MEL50 and H-MEL200 variants. Thus, once again our group confirmed that melatonin has positive influence on SOD activity, as it was shown previously in cucumber seeds (Bałabusta et al., 2016). On the other hand, in our experiment no spectacular changes in the activity of the other AOX enzymes, such as: CAT, POX, APX, etc., which regulate the H_2_O_2_ status, were observed under the influence of melatonin.

The current findings suggest that seed priming fluid supplemented with melatonin enhances oxidative stress tolerance in growing plants. This beneficial effect was reflected by reduced accumulation of $O_{2}^{•–}$ in leaf tissues, probably due to increased SOD activity; preservation of photosynthetic pigments; improved functioning of the photosynthetic apparatus and higher water content in the tissues during PQ-mediated stress. Although detailed molecular mechanisms of melatonin action still need elucidation, these findings provide evidence for the physiological role of this molecule applied during seed priming and serve as a platform for its possible applications in agricultural or related areas of research.

## Author Contributions

KS: Work conception, all experiments concerning pea seeds and seedlings realization, data acquisition and analysis, drafting of the manuscript. RR: Research consultation/discussion, manuscript revision: language and editorial corrections. MP: Methodological consultant, statistical calculations, data analysis, and interpretation, manuscript revision.

## Conflict of Interest Statement

The authors declare that the research was conducted in the absence of any commercial or financial relationships that could be construed as a potential conflict of interest.

The reviewer D-XT declared a shared affiliation, though no other collaboration, with one of the authors RR to the handling Editor, who ensured that the process nevertheless met the standards of a fair and objective review.
